# Data supporting beta-amyloid dimer structural transitions and protein–lipid interactions on asymmetric lipid bilayer surfaces using MD simulations on experimentally derived NMR protein structures

**DOI:** 10.1016/j.dib.2016.03.015

**Published:** 2016-03-10

**Authors:** Sara Y. Cheng, George Chou, Creighton Buie, Mark W. Vaughn, Campbell Compton, Kwan H. Cheng

**Affiliations:** a Department of Physics, University of Texas at Austin, Austin, TX 78712, USA; b Department of Chemical Engineering, Texas Tech University, Lubbock, TX 79409, USA; c Department of Computer Science, Trinity University, San Antonio, TX 78212, USA; d Department of Physics, Texas Tech University, Lubbock, TX 79409, USA; e Department of Physics and Astronomy, Trinity University, San Antonio, TX 78212, USA

**Keywords:** Asymmetric lipid membranes, Protein structure on surfaces, Protein–lipid interactions, Protein aggregation, Beta-Amyloid

## Abstract

This data article supports the research article entitled “Maximally Asymmetric Transbilayer Distribution of Anionic Lipids Alters the Structure and interaction with Lipids of an Amyloidogenic Protein Dimer Bound to the Membrane Surface” [1]. We describe supporting data on the binding kinetics, time evolution of secondary structure, and residue-contact maps of a surface-absorbed beta-amyloid dimer protein on different membrane surfaces. We further demonstrate the sorting of annular and non-annular regions of the protein/lipid bilayer simulation systems, and the correlation of lipid-number mismatch and surface area per lipid mismatch of asymmetric lipid membranes.

**Specifications Table**

**Table:** 

Subject area	*Physics*
More specific subject area	*Membrane Biophysics*
Type of data	*Text, Graph and Figure*
How data was acquired	*Molecular Dynamics (MD) Simulations from Initial Experimental Structures*
Data format	*Procedures and Analyzed data*
Experimental factors	*Construction of dimer from two NMR PDB structures (2BEG and 1BA4)*
Experimental features	*Initial experimentally derived PDB structures used to perform MD simulations*
Data source location	*San Antonio, Texas, USA*
Data accessibility	*Data are supplied in this article*

**Value of the data**

• Membrane surface kinetics and time evolution of secondary structures are useful parameters to study amyloidogenic proteins
• Residue contact maps can be used to quantify domain aggregation of proteins on lipid membrane surfaces
• Sorting lipids into annular and non-annular regions can be used to identify local and longer-range effects of protein lipid interactions.
• Surface area per lipid versus lipid number mismatch plots can be useful when estimating the number of lipids to include in constructing lipid bilayer systems.

## 1 Data

The data provides a variety of analysis methods to characterize protein–lipid interactions. These analysis methods used can be applied to any protein-lipid system, and can provide detailed information about protein structural changes over time when in contact with lipid membrane surfaces of differing composition. The design of the initial structures of the dimer protein and lipid bilayer systems can be found in the *Material and Methods* section of the research article [1].

## 2 Experimental design, materials and methods

**Fig. 1:**
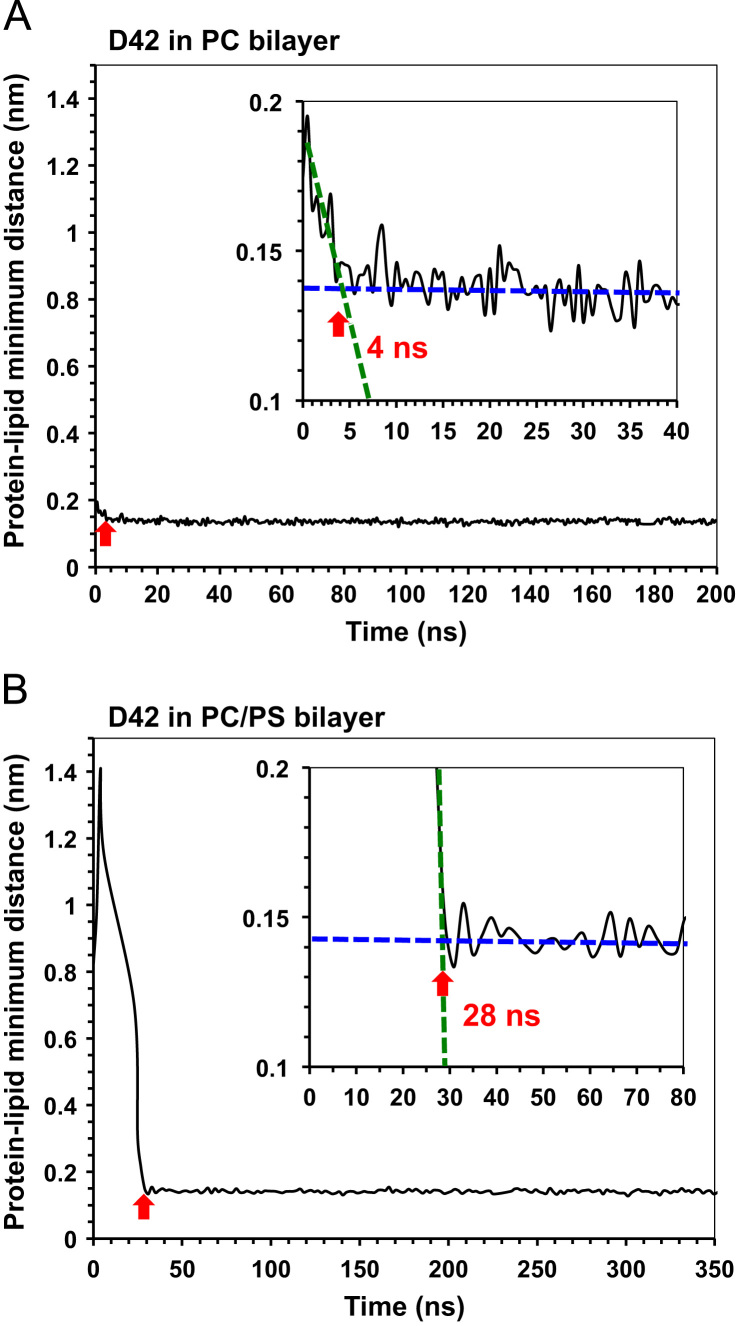
Binding kinetics of D42 on membrane surfaces. Plots of protein-lipid minimum distance between the atoms of D42 and the PC lipid in the protein-contact PC leaflet versus simulation time of two representative simulation replicates: rep1 of the PC (A) and rep1 of the PC/PS (B) bilayer systems, are shown. An enlarged surface-binding kinetics plot is shown in the inset. Each inset shows two extrapolated lines demonstrating the sharp decline (dotted green line) and the near horizontal, stabilized value around 0.14 Å (dotted blue line) of the protein-lipid minimum distance before and after the protein attachment on the PC leaflet. The initial protein attachment time, 4 ns for the PC bilayer (A) or 28 ns for the PC/PS bilayer (B) system, of protein surface binding is defined as the time of the intersection (red arrow) between the two extrapolated lines given in the inset.

**Fig. 2:**
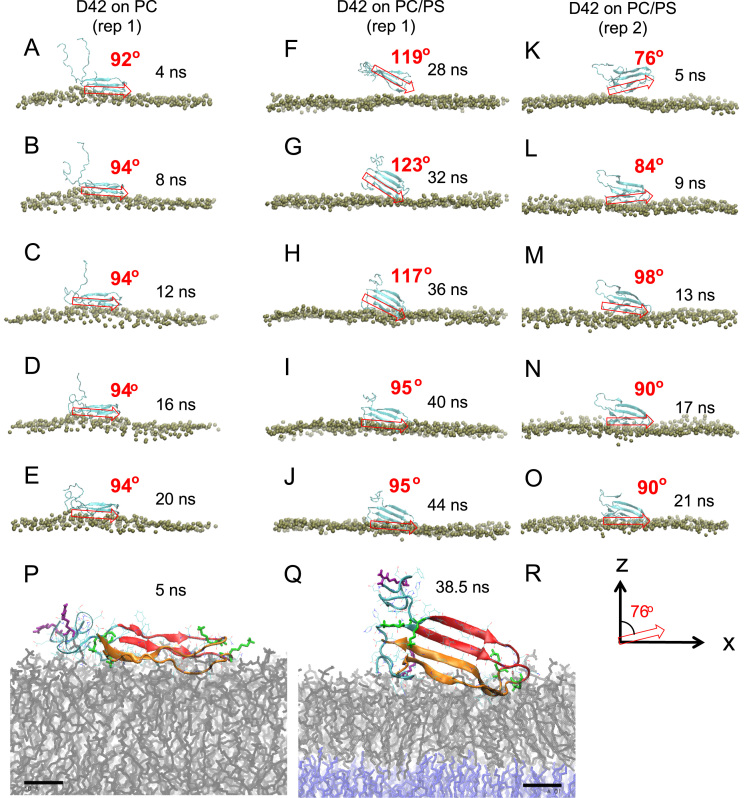
Attachment behavior of D42 on membrane surfaces. Time-dependent behavior of the membrane- orientation of D42 after the initial protein attachment (see Fig. 1) of three representative simulation replicates: rep1 of the PC (A–E), rep1 of the PC/PS (F–J), and rep2 of the PC/PS (K–O) lipid bilayer systems, at selected simulation times is shown. For clarity, only the phosphate atoms (silver) of the protein-contact PC leaflet and D42 (blue ribbon) are presented. A red arrow, starting from the last residue Ala-42 and ending at Lys-28 at the tip of the loop of the U-shaped chain A of D42, represents the membrane-orientation vector of the surface-contact region of D42 (see *Materials and Methods* of the research article [1]. The angle between the red arrow and the *z*-axis (as demonstrated in panel R), defined as the orientation angle, is given in red at each selected time as the protein establishes stable surface-contact with the PC leaflet during the protein surface-absorption on the lipid membrane. The representative transverse structures of D42 at 5 ns for simulation replicate rep1 of the PC (P) and 38.5 ns for simulation replicate rep1 of the PC/PS (Q) bilayer systems are shown. The *N*-terminal domain (residues 1–17 in blue ribbon) and *C*-terminal domain (residues 18–28 in red and residues 29-42 in orange) of D42 are shown. The six positively charged residues (lysine in green and arginine in purple) are in thick licorice lines, and other residues are in thin color line. A scale bar of 10 Å is given.

**Fig. 3:**
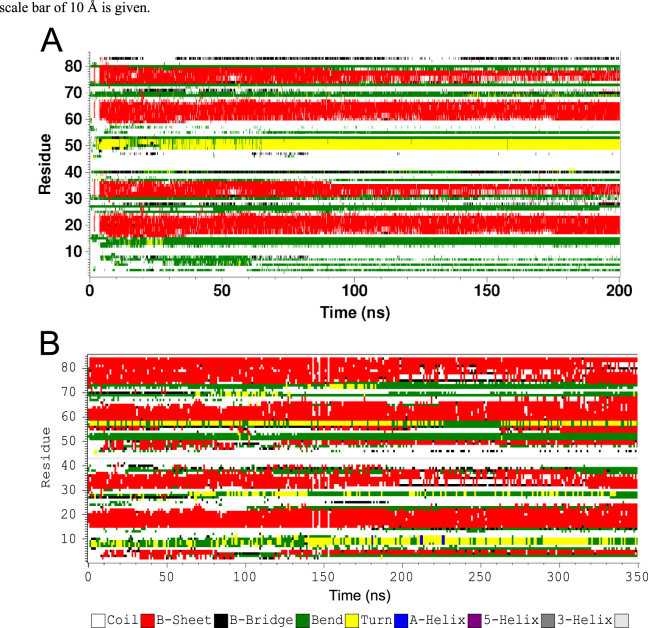
Time evolution of secondary structure of D42 on membrane surfaces. Plots of secondary structure of each residue of D42 on the protein-contact PC leaflet versus simulation time of two representative simulation replicates: rep4 of the PC (A) and rep2 of the PC/PS (B) lipid bilayer systems, based on the DSSP algorithm (see Table 1 and *Materials and Methods* of the research article [1]). In the three-dimensional plots, the residue locations of chain A and chain B of D42 are identified as residues 1–42 and residues 43–84, respectively, along the vertical *y*-axis, the time is along the horizontal *x*-axis, and 9 possible secondary structures of each residue (*z*-dimension) are color-coded.

**Fig. 4:**
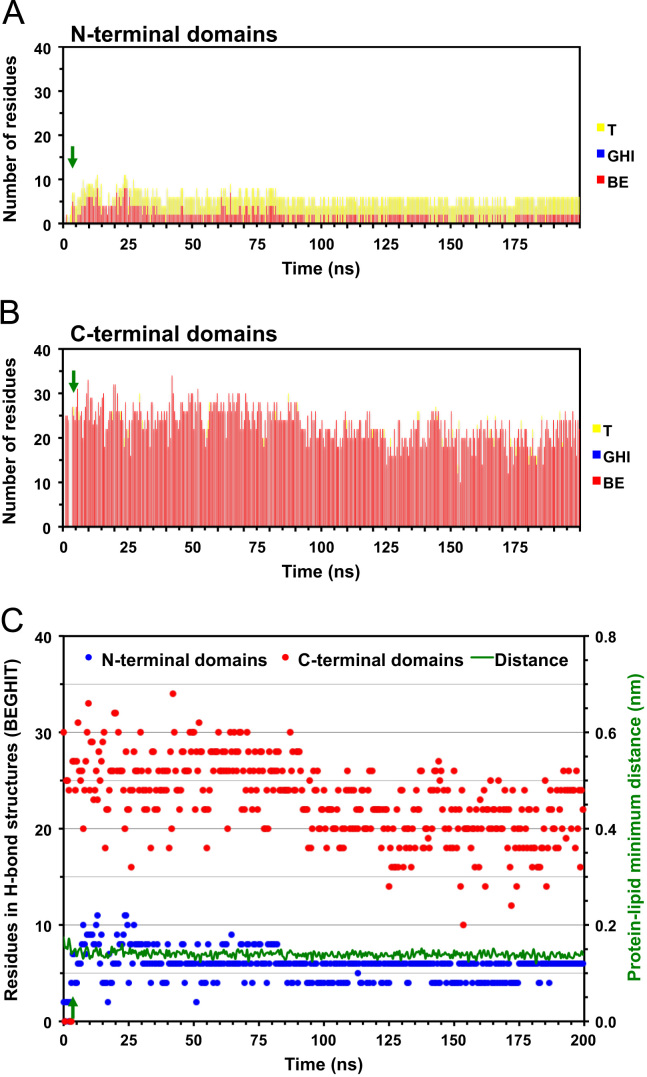
Time evolution of reduced secondary structure of D42 on the symmetric PC membrane surface. Plots of the number of residues participated in reduced secondary structures of D42 on the protein-contact PC leaflet of the symmetric PC bilayer system as a function of simulation time for simulation replicate rep1 (See Table 1 and *Materials and Methods* of the research article [1]). The numbers of residues participated in hydrogen-bonded, or H-bonded, (BE, GHI and T) structures in the *N*-terminal (A) and *C*-terminal (B) domains of D42 are presented in cumulative histograms. The sum of all hydrogen-bonded structures (BEGHIT) and the protein–lipid minimum distance versus time plots (C) are also shown. The green arrow indicates the initial attachment time at 4 ns (see Fig. 1 and Table 1 of the research article [1]) of D42 on the protein-contact PC leaflet.

**Fig. 5:**
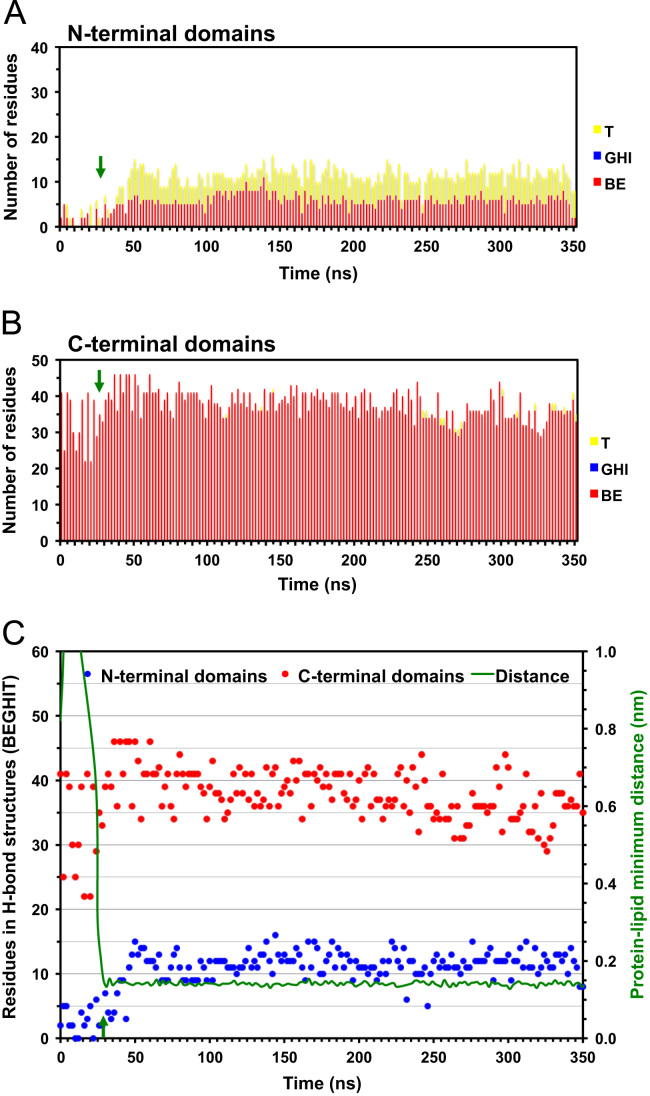
Time evolution of reduced secondary structures of D42 on the asymmetric PC/PS membrane surface. Plots of the number of residues participated in reduced secondary structures of D42 on the protein-contact PC leaflet of the asymmetric PC/PS bilayer system as a function of simulation time for simulation replicate rep1 (See Table 1 and *Materials and Methods* of the research article [1]). The green arrow indicates the initial attachment time at 28 ns (see Fig. 1 and Table 1 of the research article [1]) of D42 on the protein-contact PC leaflet. See the legend of Fig. 4 for other details.

**Fig. 6:**
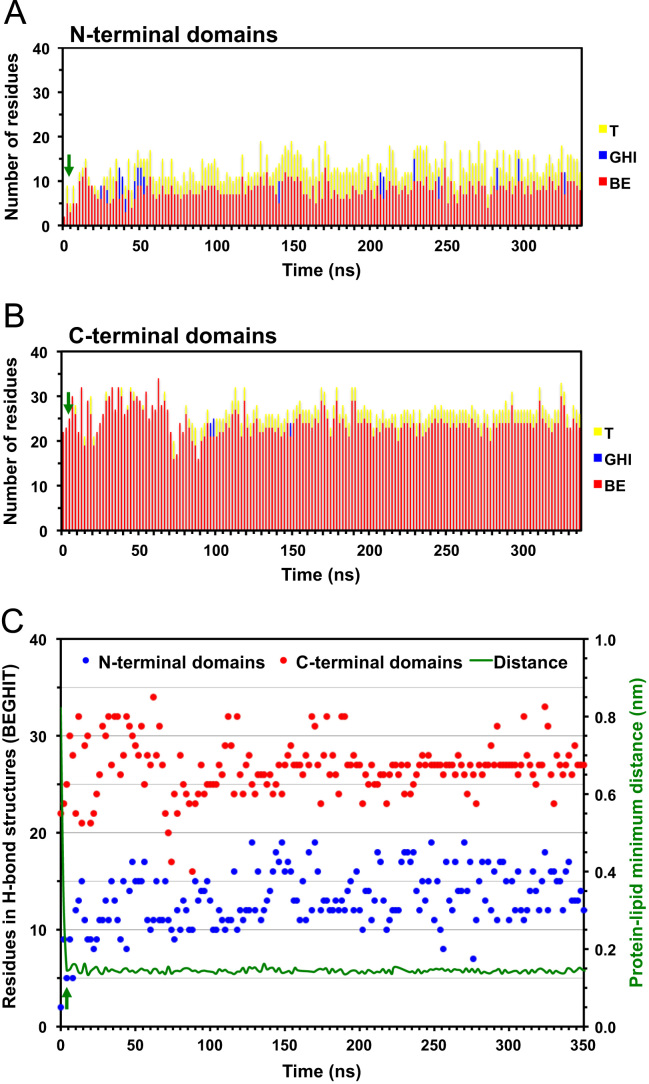
Time-evolution of reduced secondary structures of D42 on the asymmetric PC/PS membrane surface. Plots of the number of residues participated in reduced secondary structures of D42 on the protein-contact PC leaflet of the asymmetric PC/PS bilayer system as a function of simulation time for simulation replicate rep2 (See Table 1 and *Materials and Methods* of the research article [1]). The green arrow indicates the initial attachment time at 5 ns (see Table 1 of the research article [1]) of D42 on the protein-contact PC leaflet. See the legend of Fig. 4 for other details.

**Fig. 7:**
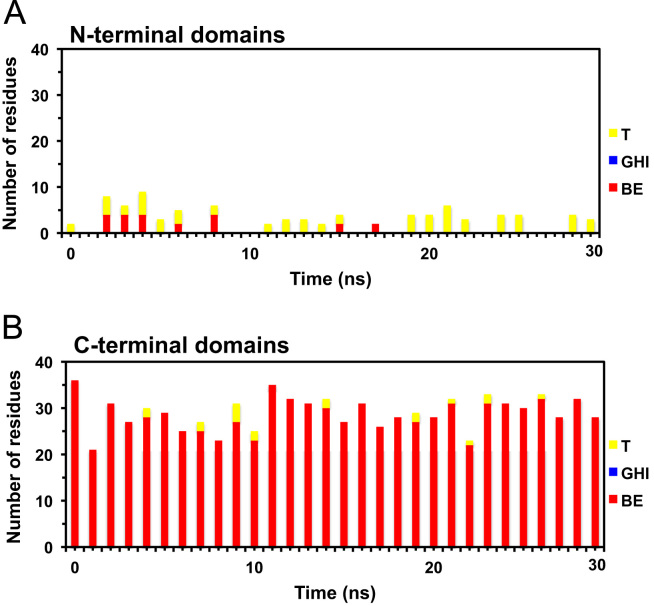
Time evolution of the reduced secondary structures of D42 in solution. Plots of the number of residues participated in reduced secondary structures of D42 in solution as a function of simulation time. See the legend of Fig. 4 for other details.

We provide an analysis of a human beta-amyloid protein D42 (dimer of DAEFRHDSGYEVHHQKLVFFAEDVGSNKGAIIGLMVGGVVIA) as well as some comparisons with the beta-amyloid D40 (same sequence less the 2 terminal amino acids IA) [2]. The initial protein structures of D42 and D40 were derived from published NMR experiments, i.e., PDB structures 2BEG [3] and 1BA4 [4]. We investigate structure formation and evolution [5] and binding kinetics on a symmetric 1-palmitoyl-2-oleoyl-phosphatidylcholine (PC) single bilayer system and an asymmetric 1-palmitoyl-2-oleoyl-phosphatidylcholine/1-palmitoyl-2-oleoyl-phosphatidylserine (PC/PS) double bilayer system (Fig. 1, Fig. 2, Fig. 3, Fig. 4, Fig. 5, Fig. 6, Fig. 7). We also provide residue contact-maps of the dimer on the symmetric and asymmetric bilayer systems (Fig. 9, Fig. 10, Fig. 11).

**Fig. 8:**
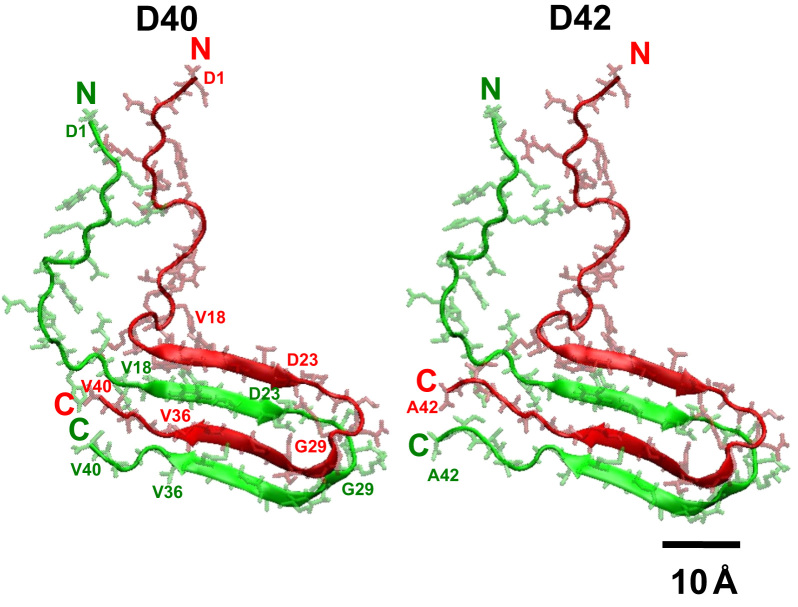
Comparison of initial structures of two beta-amyloid dimer isoforms. Two beta-amyloid dimer isoforms, D40 and D42, are shown. The sequence of D40 is derived from that of D42 by removing the last two amino acids in the *C*-terminal domain. Therefore, D40 has a C-terminus of V40 as shown. The initial structure of D42 is the same as given in Fig. 1 of the research article [1]. The top (chain B) and bottom (chain A) monomers are labeled in red and green, respectively. Protein residues are shown in lines, and the backbone secondary structures in ribbons. A scale bar of 10 Å is given.

**Fig. 9:**
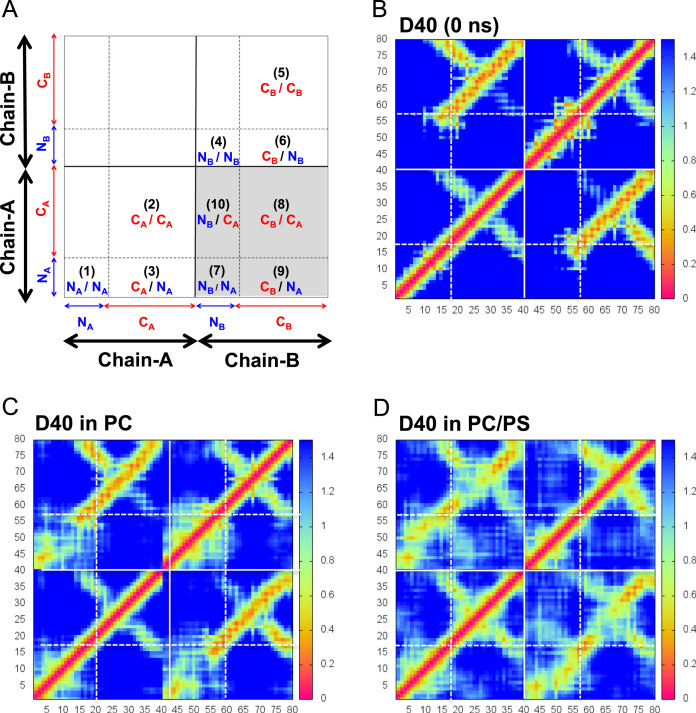
Residue-contact maps of D40 on membrane surfaces. Protein residue-contact maps of the beta-amyloid dimer D40 before (B) and after absorption on the protein-contact PC leaflets of the PC (C) and PC/PS (D) bilayer systems are shown. The maps are in the form of three-dimensional minimum distance (color-coded in the *z* dimension) versus residue location. The residue location is in a two-dimensional residue location-matrix on the *x*–*y* plane. A zone-directory map (A) identifies the zones of minimum distances among protein residues in the *N*-and *C*-terminal domains from the same chain, A-A (1–3) and B–B (4–6), and from different chains, A and B (7-10 shaded in gray). Here, N_A_ and C_A_ refer to the *N*-and *C*-terminal domains of chain A, and N_B_ and C_B_ refer to the *N*-and *C*-terminal domains of chain B. The residue-contact maps before the simulation or 0 ns (B), and averaged over the last 50 ns and across all repeating replicates in the PC (C) and PC/PS (D) bilayer systems are given. The vertical color bar besides each residue-contact map (B, C or D) shows the minimum distance scale among residues in nm. See *Materials and Methods* of the research article [1] for details of generating the residue-contact maps.

**Fig. 10:**
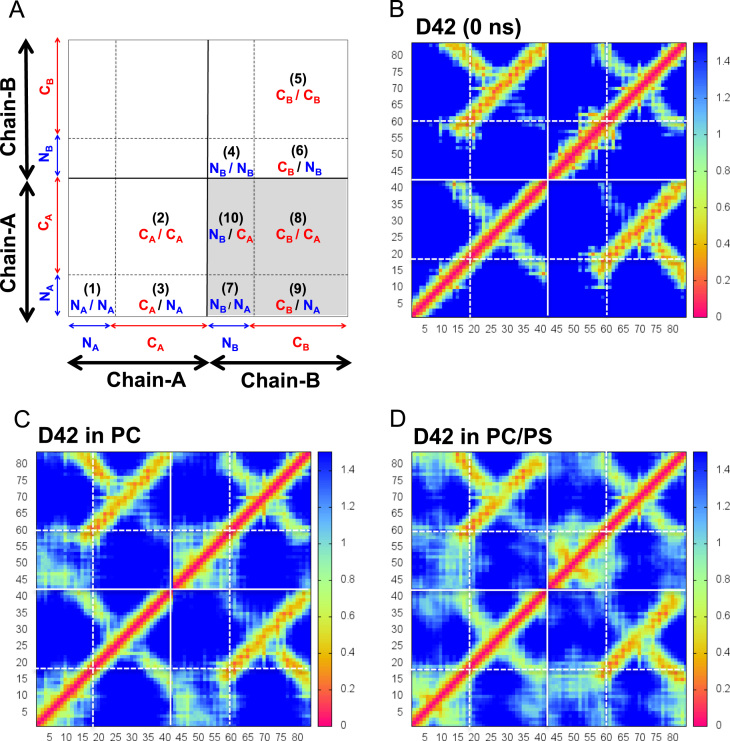
Average residue-contact maps of D42 in protein/lipid bilayer systems. See the legend of Fig. 9 for details.

**Fig. 11:**
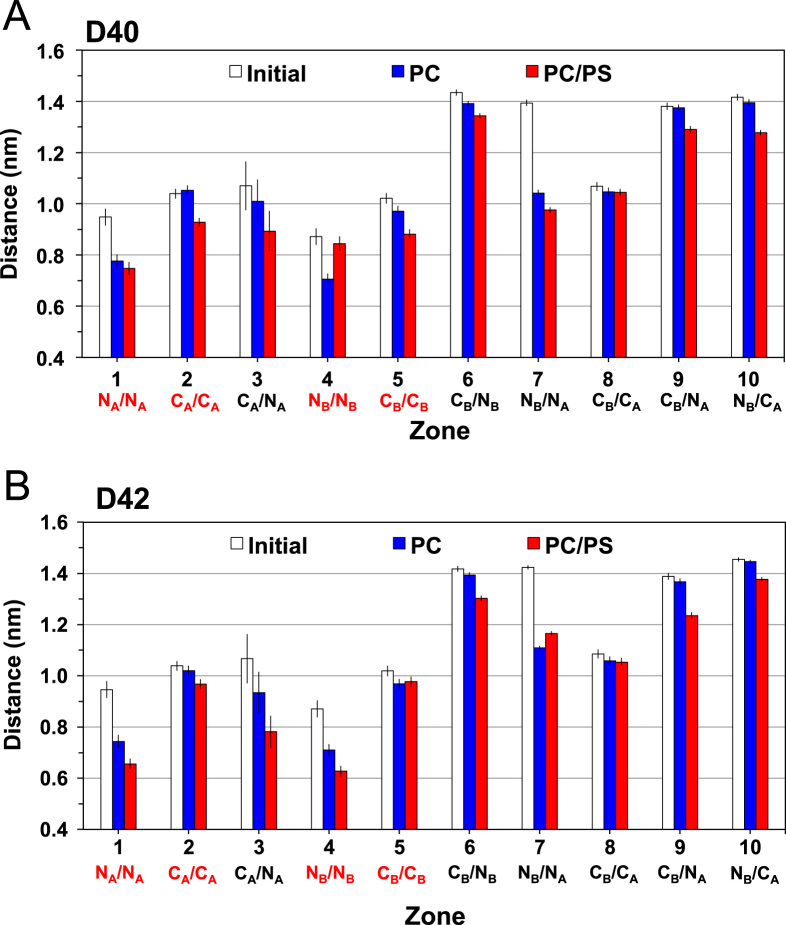
Comparison of average residue-contact distances of D40 and D42 on membrane surfaces. Average residue-contact distances of D40 (A) and D42 (B) in 10 residue-contact zones (see Fig. 9, Fig. 10) for the protein absorbed on the PC leaflets of the PC (blue) and PC/PS (red) bilayer systems are shown. The average distance was over the last 50 ns of each replicate and across all four repeating replicates, with means and standard errors of the means (error bars) given. The values before the simulations, i.e., initial or 0 ns (white), are presented for comparison. The four contact zones (N_A_/N_A_, C_A_/C_A_, N_B_/N_B_ and C_B_/C_B_) among residues within the same chain, i.e., chain A or chain B, are marked in red in the horizontal axis to facilitate the comparison. The definitions of the contact zones are given in Fig. 9. Data for D42 (B) is the same as Fig. 5A in the research article [1] and is shown here for direct comparison with the data for D40 (A).

**Fig. 12:**
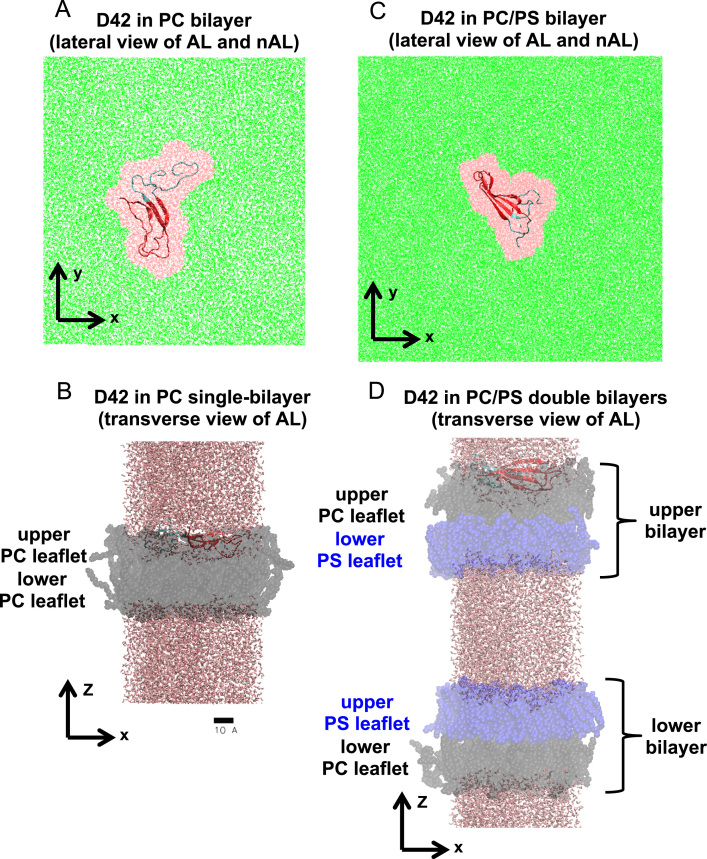
Sorting of annular and non-annular regions in a protein/lipid bilayer system based on Surround algorithm. The lateral-view or *x*–*y* plane (A and C) and transverse-view or *x*–*z* plane (B and D) of a protein (D42) in a PC single-bilayer system (A and B) and a PC/PS double bilayer system (C and D) are given. The *z*-axis is along the normal of the lipid bilayer. For the lateral-view, only the water (pink) and protein (color ribbons) within the annular region (AL), and the water within the non-annular region (nAL) are shown. For the transverse-view, lipid (PC in black and PS in blue), water (brown) and protein (color ribbons) of AL are shown. The *N*-and *C*-terminal domains of D42 are shown in blue and red ribbons, respectively. The upper and lower lipid leaflets in each lipid bilayer are shown. See *Materials and Method* of [1] for details of the Surround algorithm. A scale bar of 10 Å is shown.

**Fig. 13:**
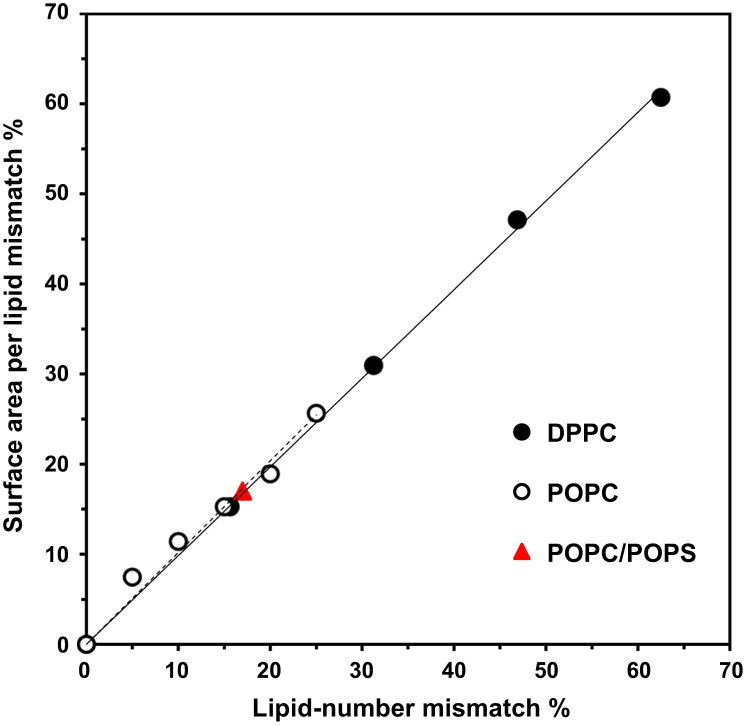
Correlation between lipid-number mismatch and surface area per lipid mismatch in asymmetric lipid membranes. A plot of surface area per lipid mismatch percentage versus lipid-number mismatch percentage in single-component dipalmitoyl-PC, or DPPC, (filled circles), single-component 1-palmitoyl, 2-oleoyl-PC, or POPC, (open circles), and two-component PC/PS, or POPC/POPS, (red triangles) simulated lipid bilayer systems are shown. The data of DPPC [10] and POPC [11] systems were extracted from previously published studies as indicated. The lipid-number mismatch percentage is defined as the difference in the lipid-numbers between two leaflets divided by the larger lipid-number from one leaflet and multiplied by 100. Similarly, the surface area per lipid mismatch percentage is defined as the difference in the surface areas per lipid between two leaflets divided by the larger surface area per lipid from one leaflet and multiplied by 100. The values of the larger lipid-number from one leaflet for the DPPC, POPC and POPC/POPS bilayer systems were 64, 160 and 576. Note that, for a lipid-number mismatch of ~63%, the surface area per lipid of a 4 times larger DPPC bilayer system generated an identical surface area per lipid mismatch percentage of ~61% as that of the smaller bilayer system [10]. Similarly, the surface area per lipid mismatch of a 9 times smaller POPC/POPS is identical to that of the POPC/POPS in this work. These observations demonstrate the system size independence of the surface area per lipid in the asymmetric membranes.

Further, we sort the lipids into annular and non-annual regions (Fig. 12). Finally, we plot the lipid-number mismatch and surface area per lipid mismatch of several asymmetric lipid membrane systems (Fig. 13). The design of the initial structures (Fig. 8) of the dimer protein and lipid bilayer systems is provided in *Materials and Methods* of the research article [1]. The data was created using in-house scripts (surround for annular and non-annular region identification, and a python script for sorting of DSSP output) [6], [7], g-tools (a suite of analysis programs available through the GROMACs MD engine [8]) for secondary structure analysis, binding kinetics, and residue contact maps, and VMD [9] for visualization of structures.
