# Supplementary material for: Data supporting beta-amyloid dimer structural transitions and protein–lipid interactions on asymmetric lipid bilayer surfaces using MD simulations on experimentally derived NMR protein structures
Source: Data Brief. 2016 Mar 10;7:658–72. doi: 10.1016/j.dib.2016.03.015 (PMC4802547; doi:10.1016/j.dib.2016.03.015)
Supplement: Supplementary file 1 — Supplementary material [file mmc1.docx]

We have no conflict of interests in this article.
